# Recurrent Episodes of Fluid Retention in a Patient with Heart Failure and Chronic Kidney Disease: The Additional Value of Implantable Monitoring Systems

**DOI:** 10.1155/2021/5122917

**Published:** 2021-10-29

**Authors:** Marc Vanderheyden, Sofie Verstreken, Richard Houben

**Affiliations:** ^1^Cardiovascular Center, Onze Lieve Vrouwziekenhuis, Aalst, Belgium; ^2^2BMedical, Maastricht, Netherlands

## Abstract

The additional role of continuous monitoring of filling pressures and impedance in heart failure patients with chronic kidney disease remains undetermined. In this case report, the effects of diuretic therapy and renal replacement therapy by hemodialysis upon right ventricular filling pressures and impedance are described in a patient with end-stage heart failure and end-stage chronic kidney disease (grade 5). We demonstrated that unloading of the heart by hemodialysis partly restored the blunted Frank-Starling relationship.

## 1. Introduction

Chronic heart failure (CHF) has emerged as a major public health problem affecting close to 12 million Europeans and North Americans each year while the health care cost of these patients almost doubled [1, 2]. Despite new and more effective pharmacological and nonpharmacological therapeutic strategies, the prognosis of CHF patients remains poor [3, 4]. Because of its progressive and unstable nature, many patients, although often affected by significant comorbidities, require multiple hospital admissions for CHF decompensation, contributing to increasing health care costs. These episodes of decompensation result from high cardiac filling pressures and concomitant volume overload [5, 6].. Therefore, effective management of heart failure is primarily aimed at reducing the filling pressures and improving ventricular performance [7].

There is growing body of evidence that continuous monitoring of the filling pressures with implantable hemodynamic monitors (IHM) plays an important role in the management of the patient with heart failure. Observational as well as randomized studies revealed that trends in monitored pressures precede hospitalization by as much as 4 days and that strategies based on these data could optimize treatment and reduce hospitalizations [8, 9]. Retrospective analysis of the COMPASS study data revealed a 36% relative risk reduction (*p* = 0.03) in time to first HF hospitalization in the patients in whom the management was guided by pressure data obtained from the Chronicle IHM (Medtronic Inc. Minneapolis MN, USA) [10]. Recently, Adamson et al. found that assessment of daily changes in estimated pulmonary artery diastolic pressure (ePAD) allowed to predict a heart failure hospitalization a median of 21 days prior to the clinical event with a sensitivity of 86% and specificity of 90% [9, 11, 12].

## 2. Case Report

We report on a 75-year-old male patient who participated in a clinical investigational prospective nonrandomized study comparing OptiVol™ intrathoracic impedance measurements (InSync Sentry™, Medtronic Inc. Minneapolis, MN, USA) to right ventricular (RV) pressure hemodynamics obtained by an implantable hemodynamic monitor (Chronicle™, Medtronic Inc. Minneapolis, MN, USA). During the first 6 months of the trial, investigators were blinded to the implanted device parameters for clinical decision-making. After 6 months, information acquired by the devices was available for clinical management [13].

The patient had a history of ischemic heart disease treated by CABG with recurrent admissions for heart failure which was associated with the development of renal insufficiency grade IV. Because of recurrent episodes of heart failure, the presence of a wide QRS complex with LBBB morphology (QRS duration: 157 msec) and first-degree AV block (PR-interval: 230 msec) on the standard ECG, episodes of nonsustained ventricular tachycardia, and an ejection fraction (EF) of 15%, he fulfilled all criteria for implantation of a cardiac defibrillator with cardiac resynchronization therapy (CRT-D) (Medtronic InSync Sentry™). Along with the CRT-D, an implantable hemodynamic monitor (IHM- Medtronic Chronicle™) was implanted in the contralateral region.

At the time of implant, the patient was in NYHA class III heart failure with a weight of 70 kg, blood pressure of 110/70 mmHg, and a heart rate of 60 bpm. The central venous pressure was elevated (jugular venous height 1/2 to the jaw), and heart auscultation revealed a mitral pansystolic murmur grade 2. Blood chemistry showed an elevated NT-proBNP level of 8070 pg/ml together with a creatinine level of 4.5 mg/dl with modified diet renal disease (MDRD) of 13 ml/min/1.73 m^2^. The patient was on optimal medical therapy with a potassium-sparing and a loop diuretic and a beta blocker (Table 1). No ACE inhibitors or angiotensin receptor blockers were prescribed because of the advanced renal dysfunction.

One month following implantation (Jan 3), the patient's hemodynamic situation improved as evidenced by the rise in pulse pressure, the drop in weight and jugular vein distention (JVD), and the disappearance of leg edema and dyspnea. This hemodynamic improvement was associated with a moderate MDRD improvement and a decrease in Nt-proBNP levels to 2509 pg/ml.

**Figure 1:**
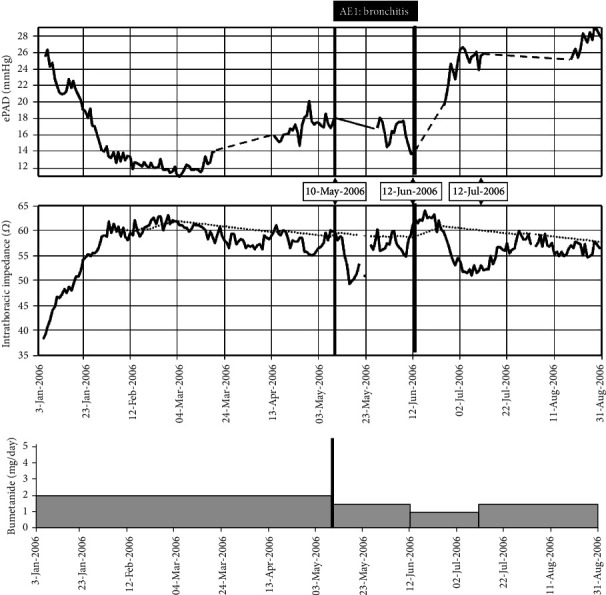
(a) Daily averaged estimated pulmonary artery diastolic pressure (ePAD). (b) Intrathoracic impedance (*Ω*) with reference line (dotted). (c) Diuretic dose (bumetanide, mg/day). AE: adverse event. Dashed lines indicate loss of long-term trend data because of remote patient hospitalization and noncompliance.

Retrospective analyses of the Chronicle and InSync Sentry™ device data confirmed this favorable response: during the first month, ePAD decreased to 12 mmHg while intrathoracic impedance increased (Figure 1, Table 1). However, five months after enrollment, the patient was hospitalized with an acute lower respiratory tract infection necessitating artificial ventilation, inotropic support, and antibiotic treatment (AE1). On admission, an increase in ePAD till 18 mmHg without major changes in impedance was noted (Table 1). Because of low blood pressures and clinical signs of dehydration, the beta blockers were temporary discontinued, and diuretics were reduced from 2 to 1.5 mg/day. As this resulted in a sudden drop of impedance from 59 *Ω* to 50 *Ω* (Figure 1, Table 1) without major change in ePAD, we speculated that the increase in lung water content secondary to the bronchitis rather than fluid overload due to heart failure accounted for this observation.

On June 12, the patient was euvolemic as evidenced by the gradual increase in impedance and decrease in ePAD. Therefore, the dose of the diuretic (bumetanide) could be further decreased to 1 mg/day and beta blockade was restarted (carvedilol; 6.25 mg/twice daily) (Figure 1). Interestingly, this resulted in a significant rise in the ePAD by 11 mmHg together with a reduction of the intrathoracic impedance, suggestive for volume overload which prompted us to adjust the diuretic regimen to 1.5 mg/day which resulted in an increase of the intrathoracic impedance to 57 *Ω* (+5 *Ω* (Table 1)).

In summary, the clinically guided adjustment of the diuretics resulted in appropriate changes in impedance and pressure with sharp fall in intrathoracic impedance, together with a gradual increase in ePAD after increase of diuretics and vice versa.

**Figure 2:**
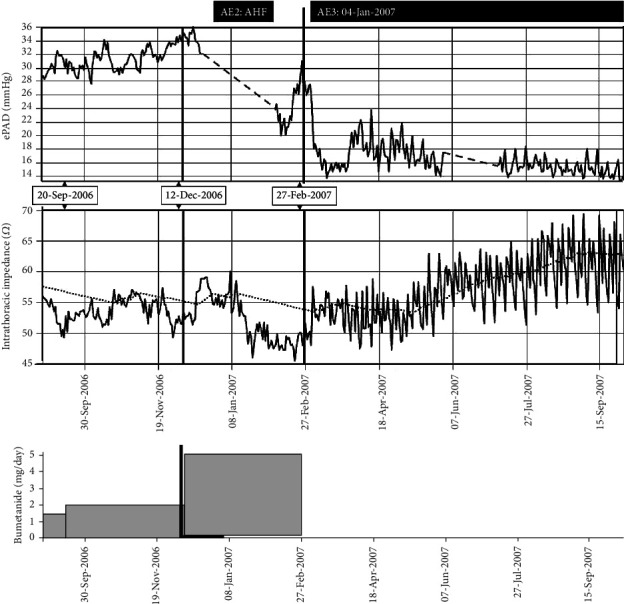
(a) ePAD (mmHg). (b) Intrathoracic impedance (*Ω*) with reference line (dotted). (c) Diuretic dose (bumetanide, mg/day). AE: adverse event. Dashed line indicates loss of ePAD data.

However, in December 7^th^, uptitration of the diuretic regimen to the initial dose (2 mg/day) because of worsening heart failure did not increase impedance and even resulted in a paradoxical increase in ePAD from 28 to 35 mmHg, together with worsening renal function and the development of acute heart failure (AE2) (Figure 2). The patient was hospitalized, the bumetanide dosage was increased till 5 mg/day, and inotropic support with dobutamine was started resulting in a transient hemodynamic improvement as evidenced by the rise in intrathoracic impedance.

Because of drug-resistant heart failure and the development of grade 5 renal insufficiency (February 27, 2007; AE3) unresponsive to diuretic therapy, the decision was taken to initiate hemodialysis three times a week. Over the following months, a typical sawtooth pattern of intrathoracic impedance and ePAD (Figure 2) was noted with higher ePAD and lower impedance the days before dialysis. Over time, there were a transient decrease in ePAD towards 15 mmHg and a corresponding increase in intrathoracic impedance from 46 to 63 *Ω* (Table 1).

## 3. Discussion

The correlation between right ventricular filling pressures, measured by the IHM and the intrathoracic fluid content, assessed by the intrathoracic impedance provides interesting insights in the complex interplay between both the heart and kidney in a patient with concomitant end-stage heart failure and kidney disease.

**Figure 3:**
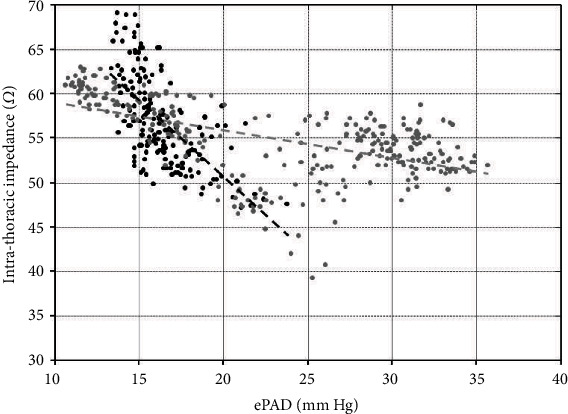
Daily averaged ePAD (mmHg) vs. intrathoracic impedance (*Z*; *Ω*). Two subpopulations of points relating ePAD to *Z* coupled to episodes before (grey) and during dialysis (black) can be identified. A significant relation (*r* = −0.56) but with only a moderate negative slope (-0.31) was found between ePAD and *Z* before dialysis (*n* = 282 days). During dialysis, a stronger negative relation was observed (*r* = −0.68; slope = −1.71, *n* = 179 days).

Of note, the much tighter coupling between ePAD and intrathoracic impedance (Figure 3) after dialysis indirectly indicates that before the initiation of the renal replacement therapy, both the failing heart and dysfunctional kidneys contributed to the rise in impedance and that dialysis prompt restored the well-known inverse interaction between ePAD and impedance.

**Figure 4:**
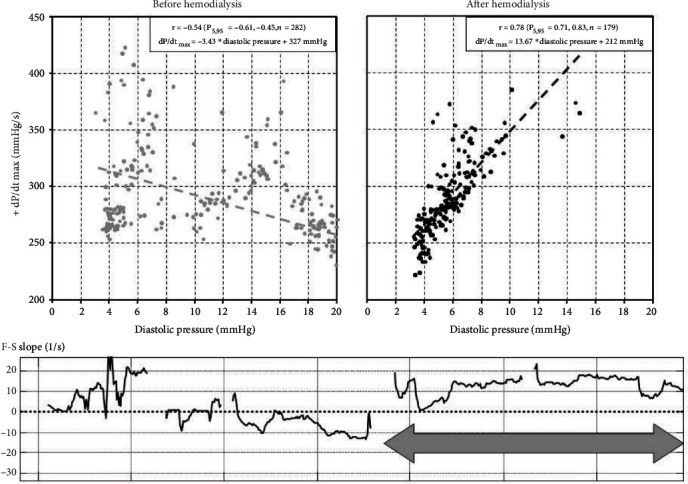
(a, b) Relation between maximum positive right ventricular dP/dt and diastolic pressure (ePAD). Two scatter diagrams show the relation between RV diastolic pressure (mmHg) and maximum dP/dt (mmHg/s) before ((a), grey) and after hemodialysis ((b), black). (c) Slope of the Frank-Starling curve obtained within a sliding window of 30 days. A positive F-S slope indicates an increase in maximum RV dP/dt (mmHg/s) at higher RV diastolic pressures (mmHg). Dashed lines indicate missing ePAD data. The arrow indicates the time frame of hemodialysis.

Furthermore, following start of dialysis, the correlation between RV dP/dt max and ePAD (Figure 4) became positive.

An inverse correlation between pressure and contractility as observed before dialysis is quite unusual in humans. However, the congestion was the result not only of impaired cardiac function but also from decreased water clearance secondary to end-stage renal failure. Therefore, we speculate that a mixture of both phenomena can explain the abnormal pressure/volume relationship. Unloading of the heart by hemodialysis favorably resulted in better hemodynamics by both a right and downwards shift in the individual Frank-starling relationship as well as by a better ventricular contractile performance ensuing in a higher dP/dt max for a similar ePAD (Figure 4).

These hemodynamic observations demonstrate that in this particular patient with both end-stage renal failure and heart failure, hemodialysis not only unloads the heart but also restores the blunted Frank-Starling response at least partially by bringing the heart back on the ascending limb of the curve and by improving its contractile performance.

## 4. Conclusion

In patients with moderate to severe HF, routine physical examination is often unable to detect early volume overload leading to overt acute heart failure episodes and costly HF hospitalizations. The continuous measurement of RV pressure and intrathoracic impedance by implantable sensors is able to prevent acute heart failure episodes, provides interesting insights in the pathophysiology, and offers a promising tool to optimize the management of these patients [9].

In this particular case, an inverse correlation between hemodynamic pressure data and intrathoracic impedance during episodes of decompensation was noted. Secondly, simultaneous monitoring of the pressure and intrathoracic impedance facilitated the correct adjustment of the HF medication and was not only able to predict but also to prevent acute HF episodes. Finally, information derived from the impedance and RV pressure measurements allowed us to unravel the close interaction between the kidney and heart in this particular patient and demonstrated how hemodialysis unloaded the heart and improved its frank-starling relationship. Long-term prospective randomized trials that establish the role of hemodynamic monitoring-guided care in patients with advanced HF and chronic kidney disease are warranted.

## Figures and Tables

**Table 1:** Clinical characteristics, lab results, medical therapy, and echocardiographic and device parameters. NYHA: New York Heart Association class; ABPsyst: systolic arterial blood pressure; ABPdiast diastolic arterial blood pressure; Creat: creatinine; MDRD: modified diet renal disease; EF: ejection fraction; MR: mitral regurgitation; TR: tricuspid regurgitation; VCI: inferior caval vein; RV: right ventricular; BiV: biventricular; Na: not available; JVP: jugular venous pressure.

	Implant	1 month postimplant	AE1: bronchitis		AE2: AHF	AE3: dialysis	
			Onset	Resolved		Start dialysis	2-month dialysis
Clinical exam							
NYHA	3	1	4	2	3	4	1
Body weight (kg)	70	67	68	67	71	73	67
Heart rate (beats/min)	60	70	80	70	70	70	70
ABPsyst (mmHg)	110	120	100	130	120	120	110
ABPdiast (mmHg)	70	80	70	70	70	70	60
JVP	Elevated	No	No	No	Elevated	Elevated	No
Leg edema	Present	No	No	No	Present	Present	No
Lung sounds/rales	Present	No	No	No	Present	Present	No
Lab results							
Creat (mg/ml)	4.50	3.80	5.25	3.53	4.50	6.00	Na
MDRD (ml/min/1.73 m^2^)	13	18	10	20	13	8	Na
Nt-proBNP (pg/ml)	8070	2509	32404	6732	29574	/	Na
Medical therapy							
Carvedilol	6.25 mg twice	6.25 mg twice	Stop	6.25 mg twice	6.25 mg twice	6.25 mg twice	6.25 mg twice
Bumetanide	2 mg	2 mg	2 mg	1.5 mg	2 mg	Dialysis	Dialysis
Echocardiography							
EF (%)	15	15	15	15	15	15	18
MR (grade)	3	2	2	2	3	3	2
TR (grade)	3	2	3	3	3	2	1
VCI	Dilated	Not dilated	Not dilated	Not dilated	Dilated	Dilated	Not dilated
RV systolic pressure (mmHg)	60	38	57	36	65	58	37
$E/\overset{´}{e}$	18	9	12	12	18	20	10
Device parameters							
% BiV pacing	/	99	93	94	96	97	94
ePAD (mmHg)	25	12	18	14	35	26	15
Impedance (Ohm)	/	60	56	62	53	46	63

## Data Availability

The data used to support the findings of this study are included within the article.
